# Programmable time-domain digital-coding metasurface for non-linear harmonic manipulation and new wireless communication systems

**DOI:** 10.1093/nsr/nwy135

**Published:** 2018-11-15

**Authors:** Jie Zhao, Xi Yang, Jun Yan Dai, Qiang Cheng, Xiang Li, Ning Hua Qi, Jun Chen Ke, Guo Dong Bai, Shuo Liu, Shi Jin, Andrea Alù, Tie Jun Cui

**Affiliations:** 1 State Key Laboratory of Millimeter Waves, Southeast University, Nanjing 210096, China; 2 National Mobile Communication Research Laboratory, Southeast University, Nanjing 210096, China; 3 Synergetic Innovation Center of Wireless Communication Technology, Southeast University, Nanjing 210096, China; 4 Photonics Initiative, Advanced Science Research Center, City University of New York, New York, NY 10031, USA; 5 Physics Program, The Graduate Center, City University of New York, New York, NY 10016, USA; 6 Department of Electrical Engineering, City College of New York, New York, NY 10031, USA; 7 Jiangsu Cyber-Space Science & Technology Co., Ltd, Nanjing 211111, China

**Keywords:** metasurface, time-domain digital coding, non-linear harmonics control, new architecture of communication

## Abstract

Optical non-linear phenomena are typically observed in natural materials interacting with light at high intensities, and they benefit a diverse range of applications from communication to sensing. However, controlling harmonic conversion with high efficiency and flexibility remains a major issue in modern optical and radio-frequency systems. Here, we introduce a dynamic time-domain digital-coding metasurface that enables efficient manipulation of spectral harmonic distribution. By dynamically modulating the local phase of the surface reflectivity, we achieve accurate control of different harmonics in a highly programmable and dynamic fashion, enabling unusual responses, such as velocity illusion. As a relevant application, we propose and realize a novel architecture for wireless communication systems based on the time-domain digital-coding metasurface, which largely simplifies the architecture of modern communication systems, at the same time yielding excellent performance for real-time signal transmission. The presented work, from new concept to new system, opens new pathways in the application of metamaterials to practical technology.

## INTRODUCTION

Non-linear electromagnetic (EM) phenomena can be observed over a wide spectrum ranging from microwave to optical frequencies [1–3]. Such phenomena are usually associated with dielectric polarization responding in a non-linear way to the incident field intensity, resulting in radiation fields oscillating at new frequencies [4–6]. Up to now, these non-linear phenomena have offered relevant functionalities for source generation, communications, optical storage and all-optical computing [7–10]. However, the mainstream methods to induce nonlinearities suffer from various problems. For example, a drawback of conventional non-linear crystals is the precise phase-matching requirements as the nonlinearly generated waves travel in the material, hindering the ability to perform elaborate wave manipulations for all harmonics [11,12]. In addition, frequency conversion requires sufficiently high optical intensities to enhance the non-linear process [13,14], which may be limited by damage thresholds in practical materials.

Recent advances of metamaterials and metasurfaces have provided an unprecedented degree of freedom to manipulate the electromagnetic waves at sub-wavelength scales, giving rise to largely enhanced nonlinearities, thus offering new possibilities to control the intensity, phase and polarization states of the induced harmonics [15–23]. Owing to the strong relation between the non-linear susceptibilities and geometrical symmetries, various asymmetric shapes of meta-atoms have been envisaged for second-harmonic generation (SHG), optimizing the element orientation as well as tuning the interaction between neighboring elements, making it possible to achieve phase control over local nonlinearities [24–27]. In addition to SHG phenomena, higher-order harmonics have also received considerable attention, such as third-harmonic generation (THG) and four-wave mixing [28–31]. However, the efficiency of frequency conversion is typically inadequate for practical applications, especially in the case of weak signals.

As an alternative approach, time-varying metasurfaces have been shown to support parametric phenomena [32–34], enabling new ways to manipulate the impinging waves in a non-linear and non-reciprocal fashion over a compact and efficient platform. Nevertheless, this approach requires an extended, often complex, modulation network, especially for arbitrary wave-manipulation schemes. A related approach to achieve non-linear control of an impinging EM wave consists of time-modulated antenna arrays [35,36]. Through a series of diodes and switches integrated into the array elements, one is able to tailor the radiation pattern at both central and harmonic frequencies, and thus benefit many microwave applications. However, this scheme relies on harmonic excitation sustained by rapid switching, thus failing to dynamically respond to external EM waves in a predefined way, limiting its applicability. Research efforts in this field have been focused on amplitude modulation of the antenna element, since complicated circuit designs are required to implement the desired phase modulation of higher-order harmonics, which inevitably increases the system complexity and the risk of performance degradation [37].

To solve the above difficulties, here we propose a new route to produce and control non-linear responses in free space, relying on a time-domain digital-coding metasurface with dynamic, programmable response to modulate the local reflection features. Inspired by spatial-domain digital-coding metasurfaces [38,39], we employ complex modulation strategies to simultaneously tailor wave–matter interactions and the frequency spectrum, where discrete reflection phase states of the metasurface are controlled with digital-coding sequences. We demonstrate non-linear processes enabled by the temporal modulation of incident waves on the metasurface with accurate control of both amplitude and phase distributions for all harmonics, which are directly determined by the external biasing voltages. So the control circuit is much simpler in comparison to current amplitude/phase modulation systems with reduced system complexity, and the possible performance degradation can be largely avoided.

We first develop a rigorous theory to describe the non-linear phenomena involved, and then validate the theory experimentally, fabricating a sample composed of periodic coding elements loaded with varactor diodes. Driven by different combinations of output voltages from a field programmable gate array (FPGA), our metasurface can implement many functions by controlling the time-domain digital-coding states. This produces a highly dynamic time-domain programmable coding metasurface, which has powerful capabilities to generate and manipulate nonlinearities, and may trigger interesting phenomena, such as velocity illusion. As an application, we explore the implementation of a binary frequency-shift keying (BFSK) communication system, which highly simplifies classical heterodyne architectures for wireless network systems. In our BFSK system, the two basic carrier frequencies are synthesized directly via the metasurface, without using complicated mixing processes, showing relevant advantages in terms of simplicity and efficiency. The proposed concept paves the way for simplified and compact communication and radar systems from acoustic frequencies to microwaves and optics.

## TIME-DOMAIN DIGITAL-CODING METASURFACE

**Figure 1.**
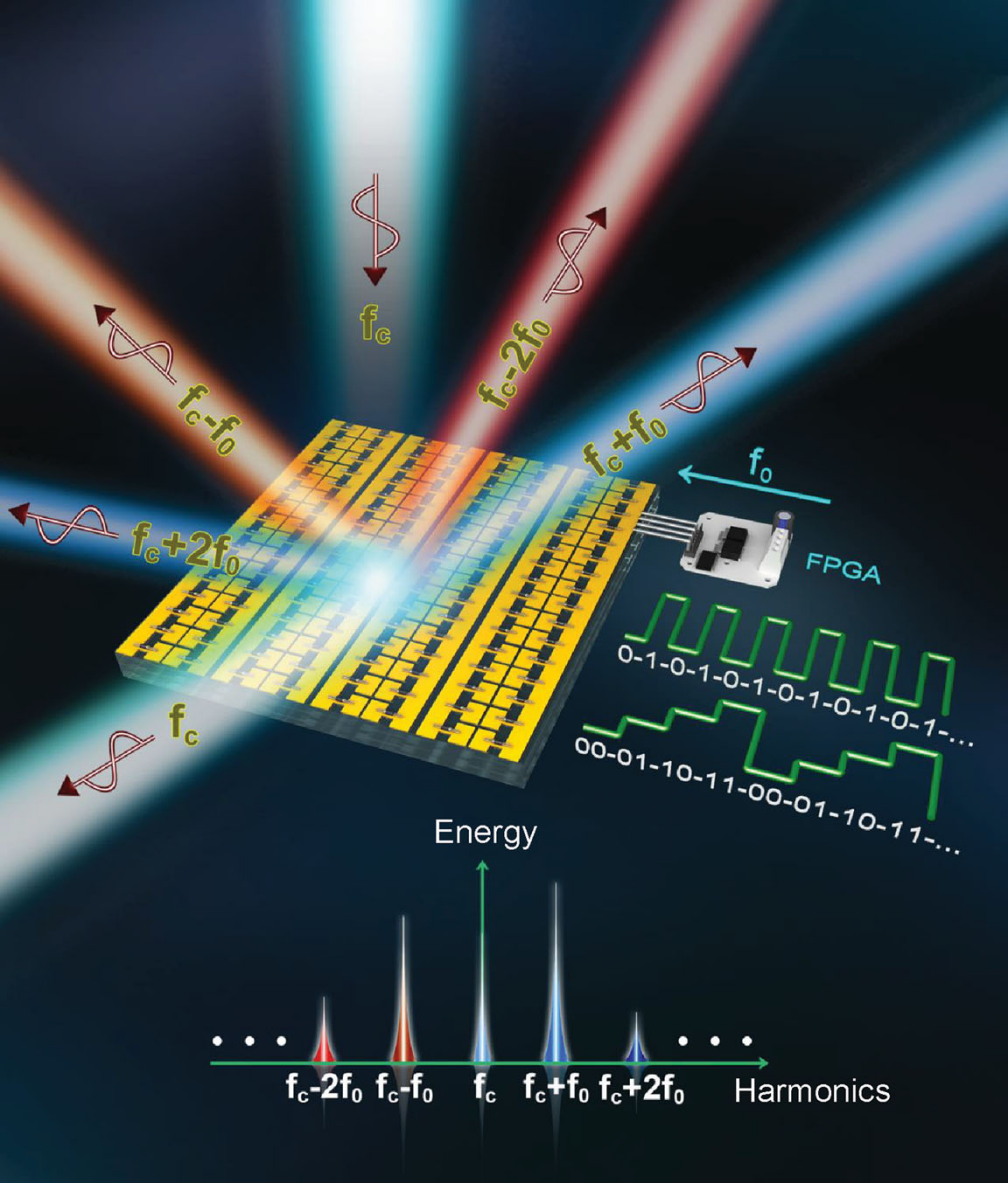
Illustration of the time-domain digital-coding metasurface. The reflection phase or amplitude of meta-atoms is electronically controlled by external biasing voltages, thus producing non-linear modulation of the spectral energies of central and harmonics frequencies by changing the coding sequence in a programmable way.

A coding metasurface is composed of digital units. For 1-bit coding, the two digital units ‘0’ and ‘1’ have either opposite phases or 0 and 1 transmission coefficients. However, the existing literature [38–43] has only studied space-domain digital-coding metasurfaces to control EM waves using different spatial-coding sequences. Here, we propose an analogue of this functionality in time-domain, in which the digital units are controlled by different periodic time sequences controlled through a FPGA, as shown in Fig. 1. We start by investigating the interaction of EM waves with the time-domain digital-coding metasurface, which is composed of periodic coding elements loaded with varactor diodes. Since the modulation has a fixed period, its reflectivity can be expressed as a periodic function of time, and defined over one period as a linear combination of scaled and shifted pulses. Under excitation with a monochromatic signal ${E_i}$($f$) with frequency ${f_c}$, the reflected signal from the time-domain digital-coding metasurface can be written as (see Supporting Online Material, SOM, available as Supplementary Data at *NSR* online):

(1) \begin{eqnarray*} {E_r}\ \left( f \right) &=& {a_0}\ {E_i}\left( f \right)+ \mathop \sum \limits_{k\ = \ 1}^\infty [{a_k}{E_i}\left( {f - k{f_0}} \right)\nonumber\\ &&+\, {a_{ - k}} {E_i}\left( {f + k{f_0}} \right)] \end{eqnarray*}

in which ${f_0} = 1/T$ (*T* is the period of the reflectivity function) and ${a_k}$ is the complex Fourier series coefficient at $k{f_0}$:

(2) \begin{eqnarray*} {a_k} &=& \frac{1}{M}\ Sa\left( {\frac{{k\pi }}{M}} \right)\exp \left( { - j\frac{{k\pi }}{M}} \right)\nonumber\\ &&\cdot \mathop \sum \limits_{m\ = \ 0}^{M - 1} {\Gamma _m}\ \exp \left( { - j\frac{{2km\pi }}{M}} \right) = UF \cdot TF\nonumber\\ \end{eqnarray*}

in which, *M* is the length of the coding sequence in one period, ${\Gamma _m}$ is the reflectivity at the interval (*m* – 1)$\tau \ $< $t < \ m\tau $, $\tau = T/M$ is the pulse width and

(3) \begin{eqnarray*} TF &=& \mathop \sum \limits_{m = 0}^{M - 1} {\Gamma _m}\ \exp \left( { - j\frac{{2km\pi }}{M}} \right),\nonumber\\ UF &=& \frac{1}{M}\ Sa\left( {\frac{{k\pi }}{M}} \right)\exp \left( { - j\frac{{k\pi }}{M}} \right). \end{eqnarray*}

It is clear that ${a_k}$ can be regarded as the product of two terms: the time factor (TF) and the unit factor (UF). The former is related to the modulation signal (${\Gamma _m}$) within different time slots, while the latter is the Fourier series coefficient of the basic pulse with pulse width $\tau $, repeated with period *T*. UF defines the basic spectrum property within one pulse, while TF represents the coding strategy. It follows that our time-domain digital-coding metasurface is able to generate a number of harmonics ${f_c} \pm k{f_0}$ by simply controlling the time-domain coding states around the central frequency. At the same time, it offers the possibility of non-linear amplitude and phase manipulation of all harmonics by tuning the coefficients ${a_k}$ using an ad-hoc modulation TF, which can be implemented in real time using the FPGA. We remark that the proposed method is different from traditional mixing techniques, as it relies on dynamic modulation of the local reflection of the coding metasurface to control and implement desired spectral features on the incident wave.

## PHASE MODULATION VIA THE METASURFACE

To illustrate the non-linear control capability on the reflected waves, we calculate the spectral distribution of all harmonics under illumination with a monochromatic plane wave. Both amplitude and phase modulation of the local reflectivity can implement efficient control of the nonlinearity. Supplementary Fig. 2, available as Supplementary Data at *NSR* online, in SOM presents the results of amplitude modulation (AM) on the metasurface. An important feature is the inherent symmetry of amplitude spectra with respect to the central frequency, due to the fact that both +*k*^th^ and –*k*^th^ Fourier series components have equal amplitude. In many applications, such as in communication systems, this feature is unwanted, since the signals may not need to be symmetrically distributed in the upper (+*k*^th^) and lower (–*k*^th^) harmonics, leading to a waste of energy and spectrum resources. At the same time, AM reflectivity is inherently required to be less than unity in some instants, hence part of the incident energy needs to be locally absorbed by the metasurface, affecting the overall efficiency. Finally, the 0^th^-order harmonic (the central frequency) cannot be totally suppressed, since the reflectivity is always positive in the whole period, which makes it impossible to realize suppression of the fundamental frequency.

**Figure 2.**
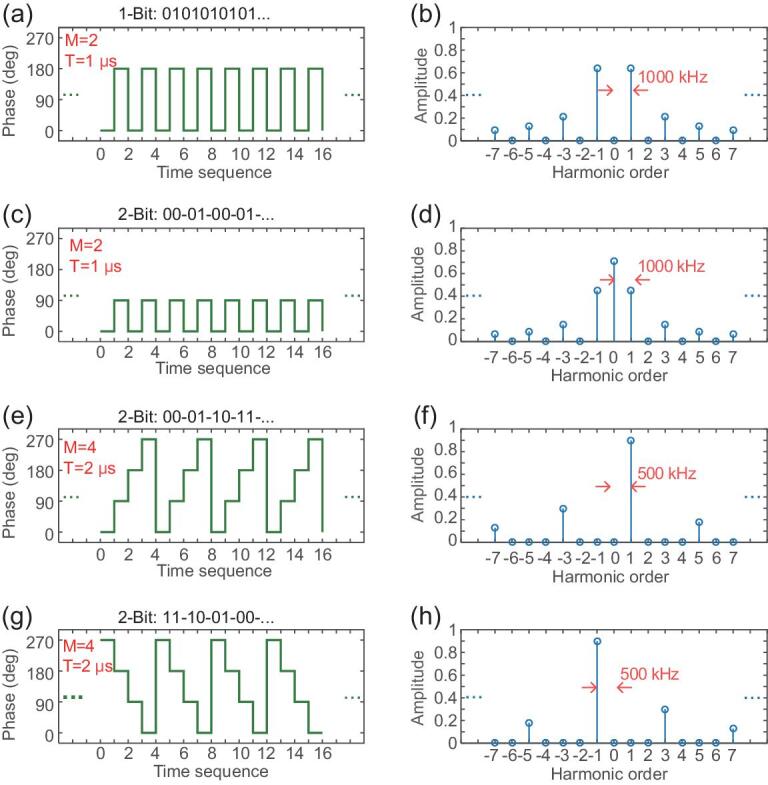
The calculated spectral intensities of the output harmonics under different PM modulations. (a) and (b) 1-bit PM coding 01010101… with *M* = 2 and *T* = 1 μs. (c) and (d) 2-bit PM coding 00-01-00-01-… with *M* = 2 and *T* = 1 μs. (e) and (f) 2-bit PM coding 00-01-10-11-… with *M* = 4 and *T* = 2 μs. (g) and (h) 2-bit PM coding 11-10-01-00-… with *M* = 4 and *T* = 2 μs.

To circumvent these disadvantages, we explore the phase modulation (PM) of the time-domain reflectivity to provide *asymmetric spectral responses* and promote modulation efficiency. Figure 2 shows the spectral intensity distribution of all harmonics excited by the metasurface under different time-coding sequences, in which the reflectivity is described as a signal with unity amplitude but digital phase states. Interestingly, we find that the 0^th^-order harmonic is totally eliminated with a 1-bit coding sequence 010101 … (*M* = 2 and *T* = 1 μs), as the reflection phase changes between 0° and 180° periodically, as shown in Fig. 2a and b. This phenomenon can be ascribed to signal cancellation in each period from anti-phase reflectivity, making the coefficient ${a_0}$ equal to zero. Figure 2c and d illustrates the case with 2-bit coding 00-01-00-01-…, in which the signals of even-order harmonics (except *k* = 0) are totally suppressed as the phase of the reflectivity switches between 0° and 90° periodically.

If more phase states are introduced in the PM signal, we can further remove unwanted harmonics and create asymmetric energy distributions in the whole spectra, as shown in Fig. 2e–h, where 2-bit coding 00-01-10-11-… and 11-10-01-00-… with anti-symmetric phase ramps is exploited to modulate the reflectivity with mirrored spectrum distributions. The reason for the observed response is that, when the reflectivity of the metasurface becomes complex and time-variant, the relationship ${a_{ - k}} = a_k^*\ $is no longer preserved in contrast to the AM scenario, resulting in spectral asymmetry. It is also easy to prove that anti-symmetric phase ramps, as in Fig. 2e and g, lead to mirror transformations, thereby translating the original signals from +*k*^th^ harmonic to the corresponding –*k*^th^ harmonic. This flexible control of harmonic spectra enabled by our time-modulated dynamic metasurface can be used to create the illusion of a system moving in space, inducing the analogue of Doppler shift, or velocity illusion. In the following experimental section, we describe in detail the operation of the metasurface for *velocity illusion* and its functionality in simplifying practical communication system architectures.

## EXPERIMENTS ON NON-LINEAR REFLECTIONS

**Figure 3.**
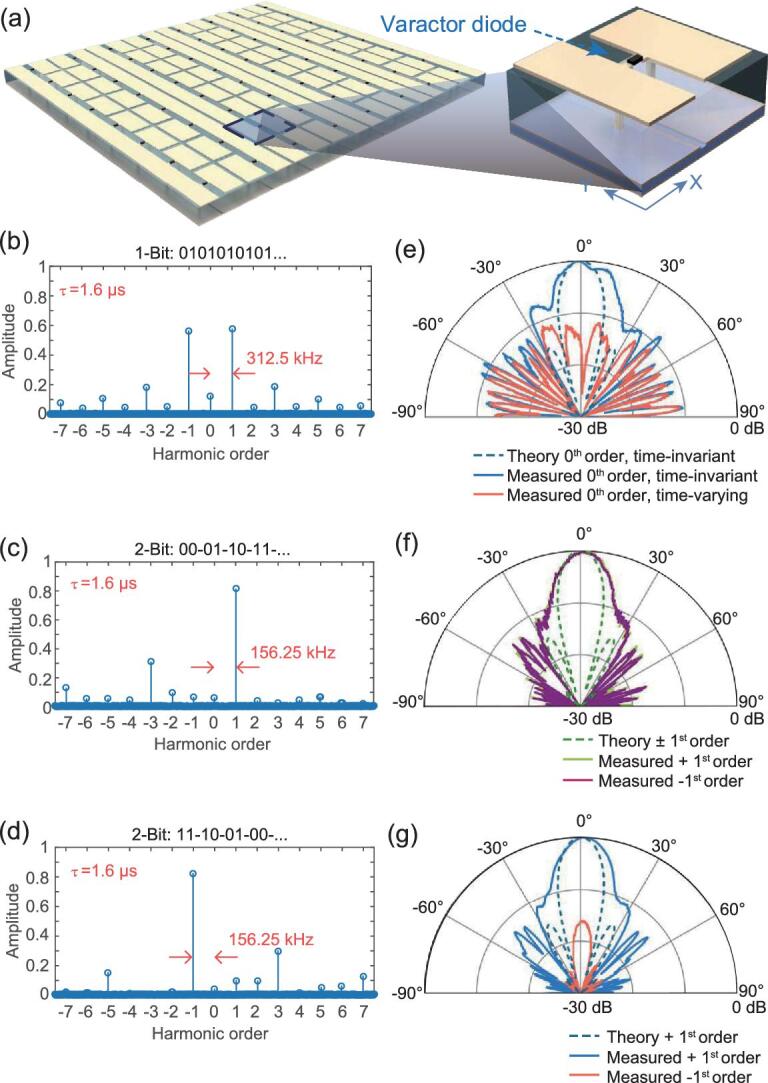
(a) Schematic of the time-domain digital coding metasurface, in which the inset shows the zoomed view of the meta-atom. (b) The measured spectral intensities of the harmonics under the 1-bit coding sequence 01010101… at 3.6 GHz with the pulse duration τ = 1.6 μs. (c) and (d) The measured spectral intensities of all harmonics under 2-bit coding sequences 00-01-10-11-… and 11-10-01-00-… at 3.6 GHz, respectively. (e) The measured H-plane scattering patterns of fundamental harmonic modulated by the 1-bit coding sequence 01010101… (blue line) or not (red line). The theoretical radiation pattern of the 0^th^-order harmonic for the time-invariant metasurface is also provided for comparison (dashed line). (f) The measured H-plane scattering patterns of the +1^st^ (the green line) and –1^st^ (the purple line) order harmonics modulated by the 1-bit coding sequence 01010101…. The dashed line shows the theoretical radiation pattern of the +1^st^-order harmonic for comparison. (g) The measured H-plane scattering patterns of the +1^st^ (the blue line) and –1^st^ (the red line) order harmonics under the 2-bit coding sequence 00-01-10-11-…. The dashed line shows the theoretical radiation pattern of the +1^st^-order harmonic for comparison.

For our experimental demonstration, we designed a PM time-domain digital-coding metasurface, as illustrated in Fig. 3a, in which the zoomed-in view of the meta-atom is shown in the inset. Two rectangular patches linked by a varactor diode (SMV-2019) are periodically repeated on top of a substrate (F4B, $\ {\epsilon _r} = 2.65( {1 - j0.001} )$ and thickness = 4 mm). A number of narrow slots (width = 0.15 mm) were etched on the bottom plate to bias the diodes through metallic vias. To eliminate EM leakage from the slots, another ultrathin vinyl electrical tape (3 M Temflex, thickness = 0.13 mm) backed by a metal layer was placed underneath the slots to prevent wave penetration. Each element has the size of 18.8 $\times $16.1 mm^2^ and the geometric dimensions are optimized to achieve continuous adjustment of the reflection phase with varied biasing voltages near 3.6 GHz. Commercial software, CST Microwave Studio, was used to quantify the spectral response of the metasurface when illuminated by a plane wave polarized in the *x* direction.

A prototype composed of 16 × 16 meta-atoms was fabricated and measured in a microwave anechoic chamber (see Supplementary Fig. 5, available as Supplementary Data at *NSR* online). Figure 3b illustrates the measured harmonics distributions modulated by the 1-bit time-domain digital coding 01010101… with two phase states (ϕ = 0° and ϕ = 180°) at different pulse durations, in which two voltages (0 V and –9 V) were chosen to bias the varactor diodes to reach ‘0’ and ‘1’ digital states. We clearly observe harmonic generation using our time-domain digital-coding metasurface, as predicted. When the pulse duration $\tau $ gradually increases from 0.8 to 6.4 μs, the harmonic energy levels are insensitive to the variation of $\tau $, since the amplitude of Fourier series is only associated with the coding sequence in modulation, as illustrated in Supplementary Fig. 6, available as Supplementary Data at *NSR* online. Consistently with the theoretical calculations shown in Fig. 2b, the proposed metasurface transfers most of the incident energy at the central frequency to higher-order harmonics. We remark that there is a small portion of residual 0^th^-order harmonic, due to simplified modelling and fabrication errors.

Figure 3e shows the measured scattering pattern of the metasurface in the H-plane for the case without modulation and with a modulation signal with binary coding sequence 01010101. As the biasing circuit is switched on, the maximum scattering directivity for the central frequency (0^th^-order harmonic) exhibits a significant drop, by 15 dB, than in the off state, corresponding to a good stealth performance. The $\pm 1$^st^-order harmonics show nearly identical radiation patterns, as a result of symmetric harmonic generation under the current coding sequence, as demonstrated in Fig. 3f.

For 2-bit coding containing four digital states ‘00’, ‘01’, ‘10’ and ‘11’, with the corresponding bias voltages 0, –6, –9 and –21 V, we achieve reflection responses with the same amplitude but discrete phase states 0°, 90°, 180° and 270°, respectively. The symmetry of the reflection spectra is broken because of the presence of time gradients. This is confirmed by the measured results in Fig. 3c, in which the $\pm $*k*^th^ harmonics display rather large intensity contrast under the periodic coding sequence 00-01-10-11-…. In particular, the harmonics of +1^st^ and –1^st^ orders have almost the same H-plane scattering pattern but make a great difference in amplitude, as shown in Fig. 3g, due to the asymmetric spectral profile. Furthermore, the modulated spectra are easily mirrored by reversing the periodic coding sequence as 11-10-01-00, as presented in Fig. 3d, since the opposite time gradient will result in the exchange of +*k*^th^ and –*k*^th^ spectral lines. Such a property can be further understood by observing the scattering pattern in Supplementary Fig. 8, available as Supplementary Data at *NSR* online, in which the $\pm $1^st^ harmonics exchange their roles. Note that we have also provided the theoretical radiation patterns in Fig. 3e and f for comparison, which agrees well with the measurement results, showing the effectiveness and correctness of the proposed method.

The large power conversion rate from carrier to first harmonics outlines the metasurface distinctive capability to realize arbitrary Doppler shift, which is especially useful for simulating echoed signals of real targets leaving the illusion of motion to deceive active radar systems, yielding velocity illusion. For instance, a motionless time-domain digital-coding metasurface with frequency shift of ±156.25 kHz (see Fig. 3c and d) may create an illusion of approaching or receding objects with velocity ±6.51 km/s to a radar operating at 3.6 GHz. By properly controlling the pulse width or coding sequence, dynamic Doppler shifts can be realized to mimic different moving targets with varied velocities using the programmable time-domain digital-coding metasurface, which enables the challenging of conventional radar systems to detect the movement of camouflaged targets.

## NEW-ARCHITECTURE WIRELESS COMMUNICATION SYSTEM

The time-domain coding metasurface shows excellent capability to embed digital signals into microwaves traveling in free space without needing external mixers, and hence may serve as an ideal platform for communication systems [44]. As shown in Figs 2e–h and 3g–h, opposite coding sequences (00-01-10-11-… and 11-10-01-00-…) enable efficient energy conversion from the central frequency to the +1^st^- and –1^st^-order harmonics, suggesting that the two spectral lines can be employed as two discrete frequencies required by traditional BFSK communication systems.

**Figure 4.**
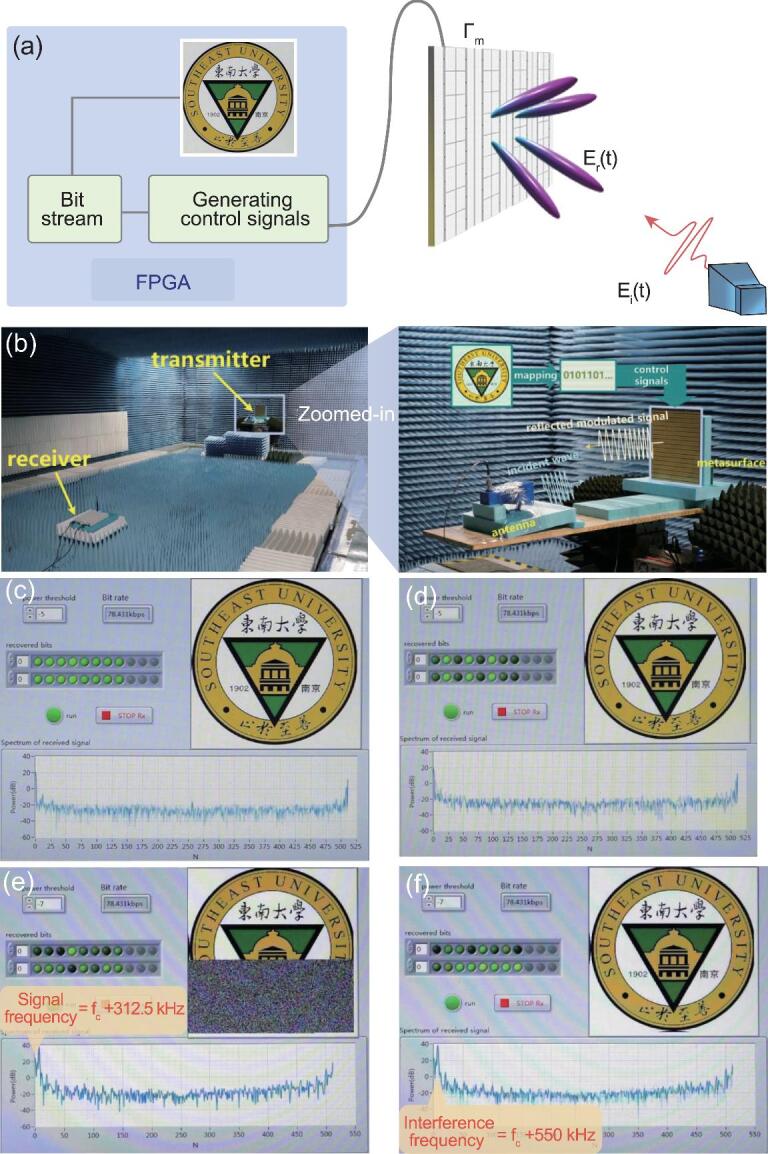
(a) Schematic of the proposed BFSK wireless communication system based on the time-domain digital-coding metasurface. (b) Experimental scenario of the BFSK wireless communication system with the transmission process described on the right. (c) and (d) The received messages by the BFSK wireless communication system for different receiving angles α = 0° and 30°, respectively. (e) and (f) Receiving process of the BFSK wireless communication system with an interference frequency at f_c_ +550 kHz, while the signal frequency is f_c_ ±312.5 kHz, to show robust anti-interference ability.

**Figure 5.**
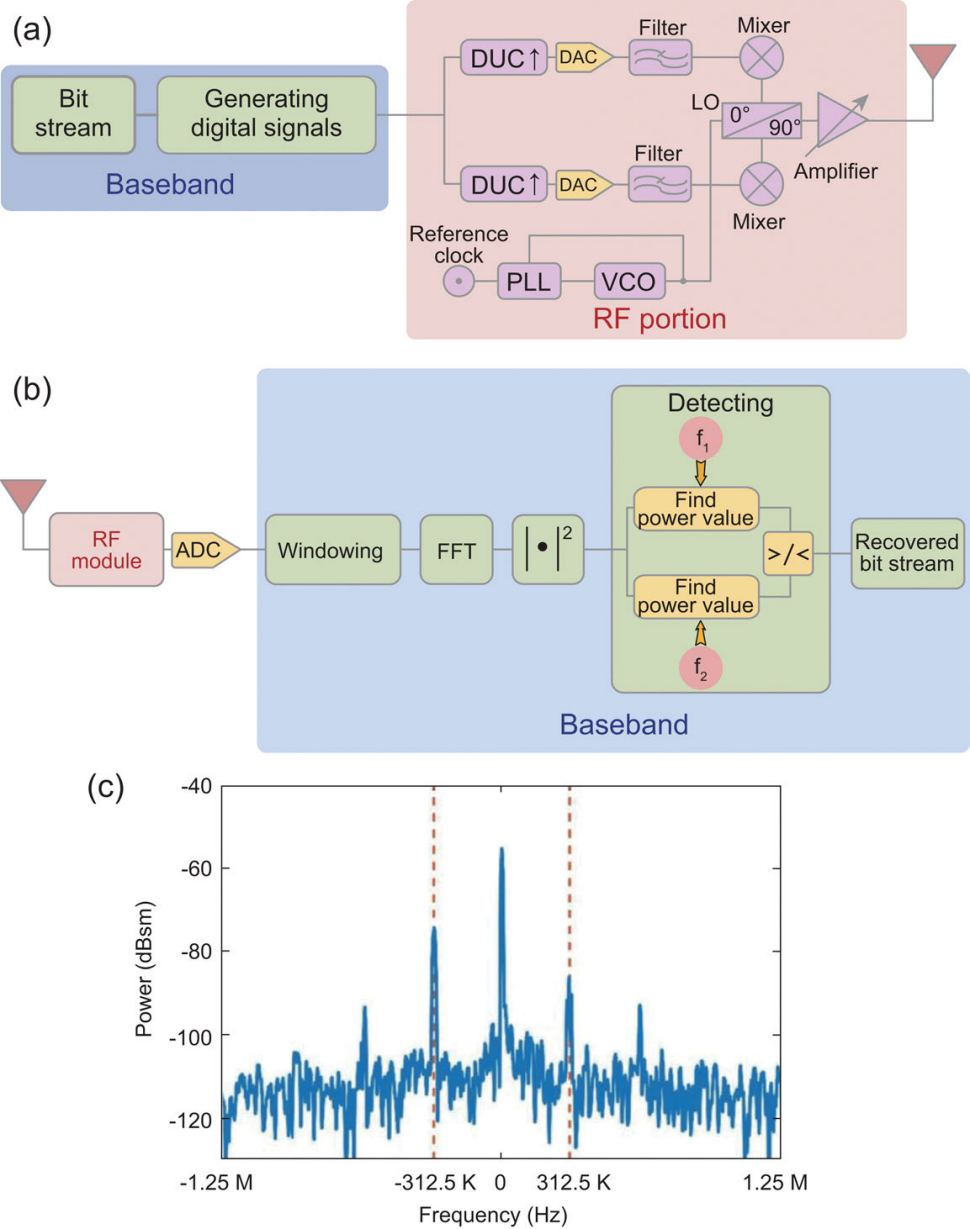
(a) The schematic of the conventional super-heterodyne wireless communication system (transmitter). (b) The block diagram of the BFSK receiver. (c) The instantaneous experimental results for the receiving spectrum after the FFT operation.

The schematic of the proposed BFSK system is illustrated in Fig. 4a, in which the time-domain digital-coding metasurface is used to replace the analogue-digital converter (ADC) and radio frequency (RF) network composed of filters, mixers and amplifiers in traditional heterodyne architecture, as shown in Fig. 5a (see Supplementary Information, available as Supplementary Data at *NSR* online, for detailed description). The real-time BFSK signal transmission is carried out from the metasurface to a soft-defined radio (SDR) receiver (NI USRP RIO 2943R). Following the conceptual diagram in Fig. 4a, the transmission process can be divided into three steps. First, the FPGA generates a bit stream (such as 01101001…) of the transmitted information (e.g. pictures and movies). Then, all bit streams are mapped to corresponding coding sequences of the metasurface, which are further extended periodically to produce a pair of discrete frequencies in BFSK. Finally, the EM waves containing the digital information are transmitted.

We built the proposed BFSK wireless communication system, as shown in Fig. 4b, in which the distance between metasurface and receiver is about 6.25 m. Figure 5b gives the block diagram of the receiver. All baseband algorithms for the BFSK receiver are performed on a SDR platform (NI USRP RIO 2943R). The time-domain signal is transformed into a frequency domain through a fast Fourier transform (FFT) operation, and is sent to the detecting diagram to determine the spectrum intensities. The upper path in the detecting diagram is responsible for energy detection of frequency offset *f*_1_ (related to bit ‘1’), while the lower path is responsible for energy detection of frequency offset *f*_2_ (related to bit ‘0’). When the power value detected in the upper path is much larger than the power detected in the lower path, the receiver judges that the current bit transmitted by the metasurface-based transmitter is ‘1’, and vice versa. After detection, the bit stream is recovered and grouped by the receiver, and conveyed for post-processing. For better illustration, Fig. 5c shows the experimental demonstration of the received spectrum after the FFT operation. The peak value is located at the lower frequency with offset of –312.5 kHz, which indicates that bit ‘0’ is transmitted in the current message symbol. The fundamental harmonic intensity in Fig. 5c is much larger than that of the $\pm {1^{{\rm{st}}}}$ order, which seems contrary to the results in Fig. 3b. Actually, the SDR receiver is used as a zero intermediate-frequency receiver, which usually leads to a problem of DC shift in practice, and contributes greatly to the large DC component in Fig. 5c. However, for the spectrum analyser, the receiving signals are sampled at the intermediate frequency, so the DC component is quite low in Fig. 3b.

Table 1 shows the key parameters of the BFSK wireless communication system based on the time-domain digital-coding metasurface. A color picture shown in Fig. 4a is successfully transmitted over the air and recovered by the SDR receiver, and the received messages are presented in Fig. 4c and d, with receiving angles α = 0° and 30° (see the definition of ${\rm{\alpha }}$ in Supplementary Fig. 5b, available as Supplementary Data at *NSR* online). The anti-interference ability of the system is tested with an interference frequency at *f*_c_ +550 kHz, which is illustrated in Fig. 4e and f. We clearly observe that, even though the receiver has a large alignment angle to the metasurface, or a strong interference frequency exists near the signal frequency, the received pictures have very good quality, verifying the feasibility and superiority of the new BFSK system. More detailed descriptions on message transmission and reception are provided in Supplementary Videos 1–3, available as Supplementary Data at *NSR* online.

**Table 1.** The primary parameters of the BFSK wireless communication system based on the time-domain digital-coding metasurface.

Parameters	Values
Carrier frequency	3.6 GHz
Sampling rate	40 MS/s
FFT size	512
Frequency offset 1 (bit ‘1’)	+312.5 kHz
Frequency offset 2 (bit ‘0’)	–312.5 kHz
Message symbol duration	12.8 μs
Bit rate of transmission	78.125 kbps

## CONCLUSIONS

We have demonstrated a new path to control nonlinearity using time-domain digital-coding metasurfaces, whose reflection phase or amplitude can be modulated periodically with the predefined coding sequences. This time-varying feature allows broad control on higher-order harmonics, enabling ad-hoc energy transfer and spectrum conversion. Different from conventional non-linear technologies, the proposed time-domain digital-coding metasurface offers substantial flexibility and accuracy in the manipulation of harmonics by simply customizing the coding sequence in a programmable way, resulting in unusual operations, like velocity illusion. Based on the time-domain digital-coding metasurface, we have proposed a novel wireless communication system with much simplified architecture. Compared to conventional systems, the hardware complexity of the metasurface-based system is greatly simplified without degrading the system performance. The metasurface-based system also holds significant potential to reduce power consumption and improves energy efficiency, and hence provides new solutions for future wireless communication systems.

## Supplementary Material

Supplemental Files
