# Supplementary material for: Programmable time-domain digital-coding metasurface for non-linear harmonic manipulation and new wireless communication systems
Source: Natl Sci Rev. 2018 Nov 15;6(2):231–8. doi: 10.1093/nsr/nwy135 (PMC8291514; doi:10.1093/nsr/nwy135)
Supplement: Supplemental Files [file nwy135_supplemental_files.zip › Final_SI_Programmable_manipulations_of_nonlinear_harmonics.docx]

Supplementary Information for

Programmable time-domain digital coding metasurface for nonlinear harmonic manipulation and new wireless communication systems

Jie Zhao^1†^, Xi Yang^2†^, Jun Yan Dai^1†^, Qiang Cheng^1,3^*, Xiang Li^2^, Ning Hua Qi^1^, Jun Chen Ke^1^, Guo Dong Bai^1^, Shuo Liu^1^, Shi Jin^2,3^*, Andrea Alù^4,5,6^, and Tie Jun Cui^1,3,7^*

**^1^**State Key Laboratory of Millimeter Waves, Southeast University, Nanjing 210096, China

**^2^**National Mobil Communication Research Laboratory, Southeast University, Nanjing 210096, China

**^3^** Synergetic Innovation Center of Wireless Communication Technology, Southeast University, Nanjing 210096, China

**^4^** Photonics Initiative, Advanced Science Research Center, City University of New York, 85 St. Nicholas Terrace, New York, NY 10031 USA

**^5^** Physics Program, The Graduate Center, City University of New York, 365 Fifth Avenue, New York, NY 10016 USA

**^6^** Department of Electrical Engineering, City College of New York, New York, NY 10031 USA

**^7^**Jiangsu Cyber-Space Science & Technology Co., Ltd., 12 Mozhou East Road, Nanjing 211111, China

† These authors contributed equally to this work.

* Corresponding author: qiangcheng@seu.edu.cn; [jinshi@seu.edu.cn](mailto:jinshi@seu.edu.cn); tjcui@seu.edu.cn

**This PDF file includes:**

Supplementary Text

Supplementary Figure S1 to S11

Supplementary Figure S1 to S4

Supplementary Equation S1 to S13

Supplementary Videos 1-3

Supplementary References

**1. Derivation of Nonlinear Modulation**

We take a reflective time-domain digital coding metasurface as example to elaborate the process of nonlinear modulation under monochromic incidence. As illustrated in Fig. S1, the reflectivity $\Gamma\left( t \right)$of the metasurface is a periodic signal, and the incidence wave is defined as $E_{i}\left( t \right)$. Thus the reflected wave $E_{r}\left( t \right)$ can be expressed by the product of $\Gamma\left( t \right)$and $E_{i}\left( t \right)$:

$$E_{r}\left( t \right)=E_{i}\left( t \right)\cdot\Gamma\left( t \right) (S1)$$

Hence the Fourier transform can be written as


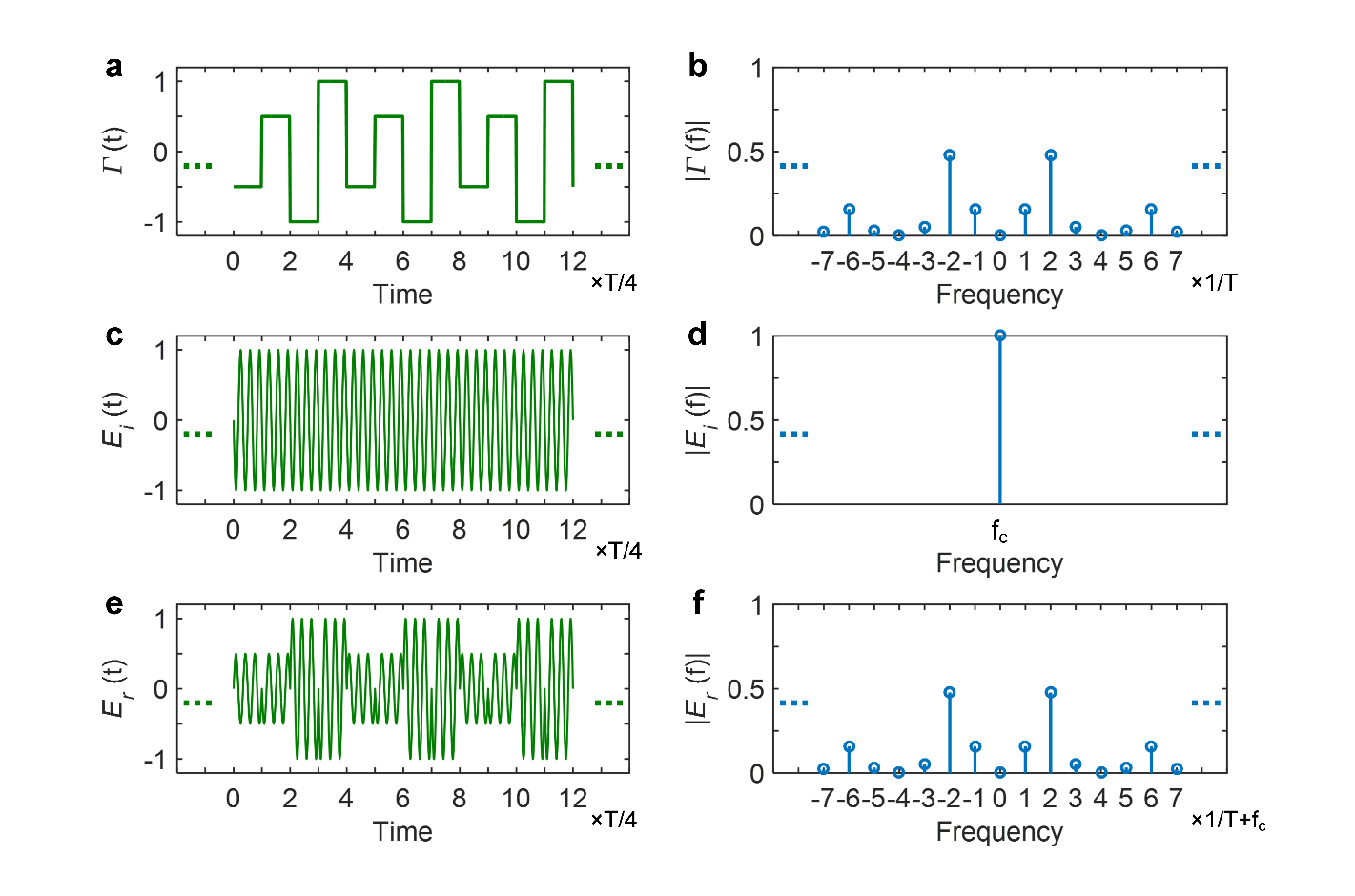


**Fig. S1 |** An example to illustrate the nonlinear modulation in the time-domain. **a-b,** The reflectivity of a time-domain digital coding metasurface and its spectrum. **c-d,** The incident waveform and its spectrum. **e-f,** The time-domain reflected waveform and its spectrum.

$$E_{r}\left( f \right)=\frac{1}{2\pi}E_{i}\left( f \right)*\Gamma\left( f \right) (S2)$$

The reflectivity function is defined over one period as a linear combination of scaled and shifted pulses

$$\Gamma\left( t \right)=\sum_{m=0}^{M-1} \Gamma_{m}g\left( t-m\tau\right), \left( 0<\left| t \right|<T \right) \left( S3 \right)$$

where $g\left( t \right)$is the periodic pulse signal, and in each period we have

$$g\left( t \right)=\left\{ \begin{aligned} 1, 0<t<\tau\\ 0, \mathrm{otherwise} \end{aligned} \right. \left( S4 \right)$$

in which *T* is the period of the reflectivity function and $\tau=T/M$ is the pulse width; *M* is a positive integer greater than zero; and $\Gamma_{m}$ is the reflectivity at the interval (*m*-1)$\tau$ <$t<m\tau$. Here the periodic pulse function $g\left( t \right)$ can be represented in alternative form of Fourier series

$$g\left( t \right)=\sum_{k=-\infty}^{\infty} c_{k}e^{jk2\pi t/T} (S5)$$

where

$$c_{k}=\frac{1}{M}Sa\left( \frac{k\pi}{M} \right)\exp\left( -j\frac{k\pi}{M} \right) (S6)$$

For a space-invariant system, the periodic reflectivity function can be represented as the sum of Fourier series

$$\Gamma\left( t \right)=\sum_{k=-\infty}^{\infty} a_{k}e^{j{k2\pi f}_{0}t} (S7)$$

and its the Fourier transform is written as

$$\Gamma\left( f \right)=2\pi\sum_{k=-\infty}^{\infty} a_{k}\delta\left( f-{kf}_{0} \right) (S8)$$

where $f_{0}=1/T,$ and $a_{k}$ is the complex Fourier series coefficient at ${kf}_{0}$. Substituting Eq. (S5) and (S6) into (S3), we have

$$\Gamma\left( t \right)=\sum_{m=0}^{M-1} \Gamma_{m}\cdot g\left( t-m\tau\right)=\sum_{m=0}^{M-1} \Gamma_{m}\cdot\left( \sum_{k=-\infty}^{\infty} c_{k}\exp\left( -jk2\pi\frac{m}{M} \right)e^{\frac{jk2\pi t}{T}} \right)$$

$$=\sum_{k=-\infty}^{\infty} c_{k}\cdot\left( \sum_{m=0}^{M-1} \Gamma_{m}\exp\left( -j\frac{2km\pi}{M} \right) \right)e^{j{k2\pi f}_{0}t} (S9)$$

Then the complex Fourier series coefficient of $\Gamma\left( t \right)$ could be expressed as:

$$a_{k}=\frac{1}{M}Sa\left( \frac{k\pi}{M} \right)\exp\left( -j\frac{k\pi}{M} \right)\cdot\sum_{m=0}^{M-1} \Gamma_{m}\exp\left( -j\frac{2km\pi}{M} \right)=UF\cdot TF (S10)$$

in which,

$$TF=\sum_{m=0}^{M-1} \Gamma_{m}\exp\left( -j\frac{2km\pi}{M} \right), UF=\frac{1}{M}Sa\left( \frac{k\pi}{M} \right)\exp\left( -j\frac{k\pi}{M} \right) \left( S11 \right)$$

It is clear that $a_{k}$ can be regarded as the product of two terms: the time factor *TF* and unit factor *UF*. The former is related to the modulation signal within different time slots, while the latter is the Fourier series coefficient of the basic pulse $g\left( t \right)$ repeated with the period *T*. It is important to mention that the time-domain reflectivity results in a frequency conversion effect on the incoming wave and introduce a series of higher harmonics at ${kf}_{0}$. Under the excitation of a monochromatic signal $E_{i}$($f$) with the frequency of $f_{c}$, the output signal can be expressed as

$$E_{r}\left( f \right)=\sum_{k=-\infty}^{\infty} a_{k}E_{i}\left( f-{kf}_{0} \right) \left( S12 \right)$$

which can be further rewritten as

$$E_{r}\left( f \right)=a_{0}E_{i}\left( f \right)+\sum_{k=1}^{\infty} {[a}_{k}E_{i}\left( f-{kf}_{0} \right)+{a_{-k}E}_{i}\left( f+{kf}_{0} \right)] \left( S13 \right)$$

**2. Amplitude Modulation**

Amplitude modulation (AM) of the reflectivity on metasurface could achieve efficient controls to the nonlinearity. Figure S2 presents the results of AM with the metasurface. We observe that the modulation function has strong impacts on the frequency offset of nonlinear components ${kf}_{0}$ and the envelope of Fourier series coefficients (or the spectrum intensities). When the surface reflectivity changes between total reflectionand total absorptionperiodically, even-order harmonics cannot be produced except for *k* = 0, as shown in Fig. S2a-b. In contrast, for the modulated reflectivity described in Fig. S2g-h, significant suppressions of high-order harmonics are obtained, which is especially beneficial to improve the generation efficiency of the second harmonic for nonlinear applications.


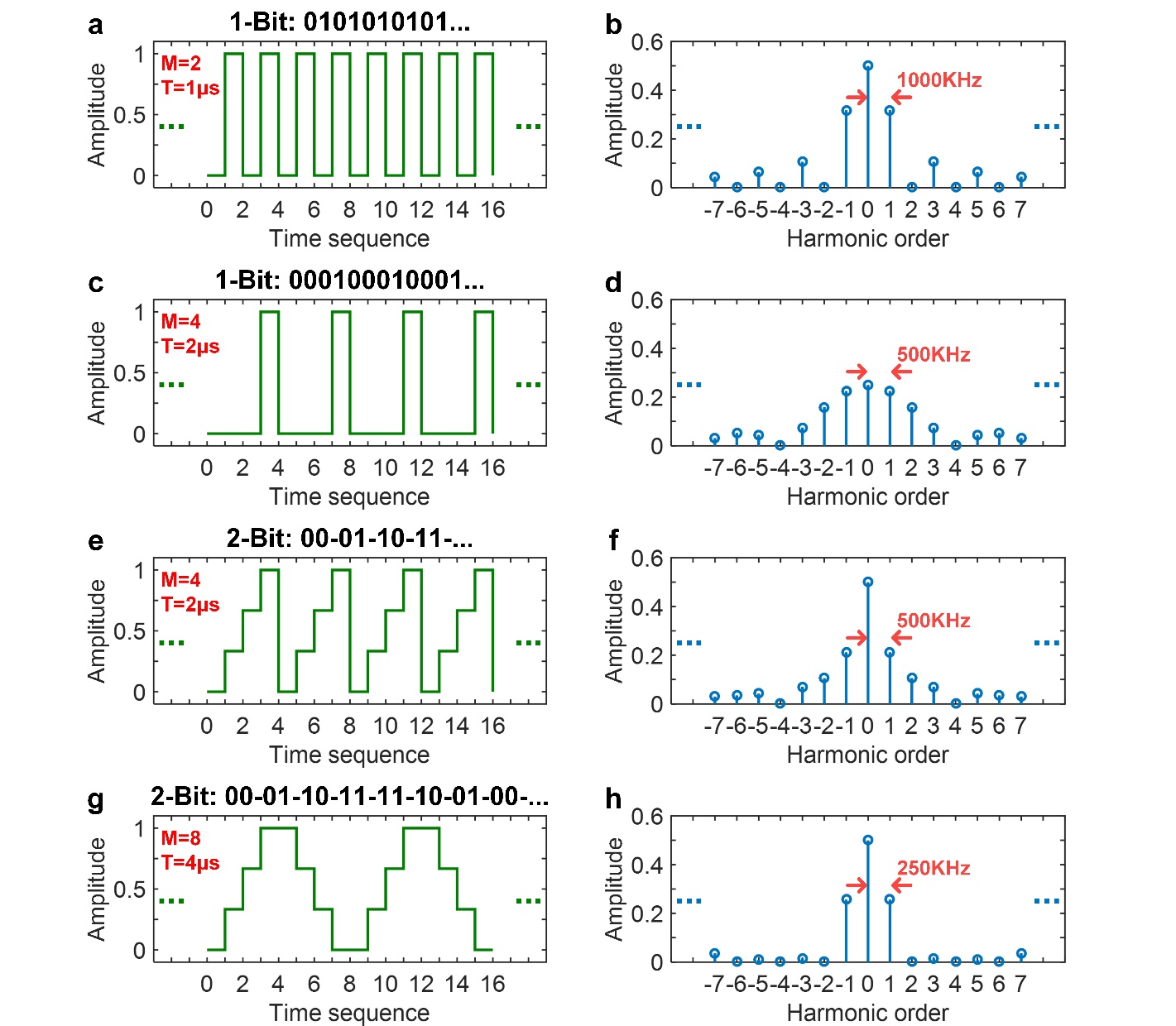


**Fig. S2 |** The calculated spectral intensities of the output harmonics under AM. **a-b,** 1-bit AM coding 01010101… with M=2 and T=1μs. **c-d,** 1-bit AM coding 00010001… with M=4 and T=2μs. **e-f,** 2-bit AM coding 00-01-10-11-… with M=4 and T=2μs. **g-h,** 2-bit AM coding 00-01-10-11-11-10-01-00-… with M=8 and T=4μs.

**3. Design of Meta-Atom**

The meta-atom of the time-domain digital coding metasurface is illustrated in Fig. S3a with its geometry details. The reflection spectra of meta-atom under different biasing voltages are simulated via the commercial full-wave simulation package CST Microwave Studio 2012. In the simulation setup, the electrical and the magnetic walls are applied in $\pm$*x* and $\pm$*y* directions of the meta-atom (see Fig. S3b), and a wave-port is employed above the structure to excite the metasurface and receive the reflected signal at the same time. The grid size is set as $\lambda$/25 at the operation frequency *f*=3.6GHz. The RLC series circuit are selected to model the varactor diode under different biasing voltages, with the effective parameters shown in Tab. S1. Figure S4a-b illustrates the simulated reflection amplitude and phase spectra as a function of the biasing voltage, showing high reflection amplitude and nearly 360° phase range in the spectra of interest, which is especially fit for PM in experiments.

**
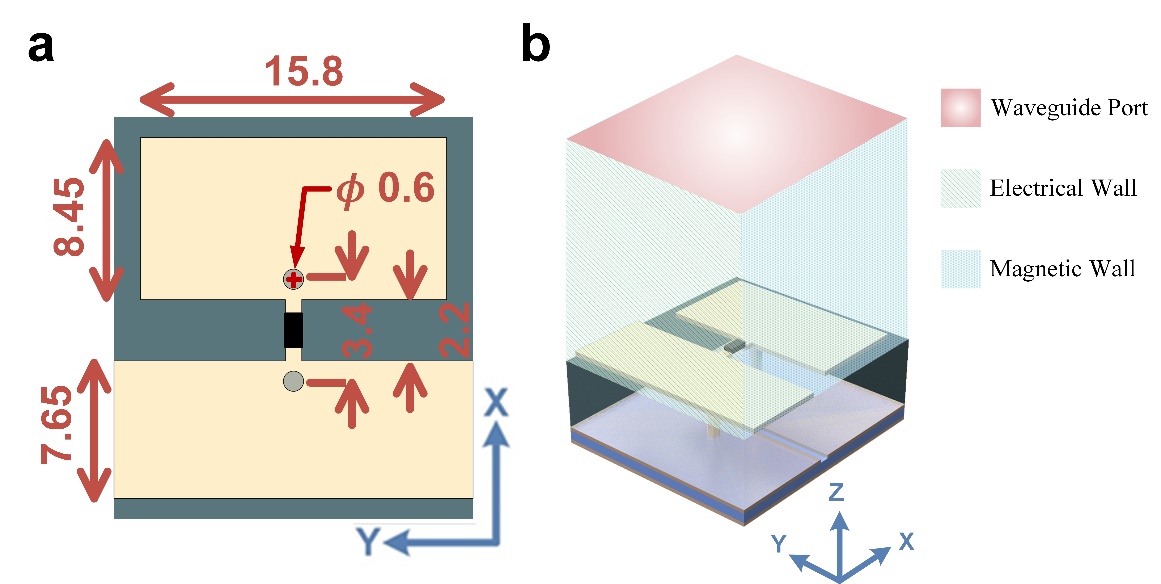
**

**Fig. S3** | **a,** Top view of the time-domain meta-atom with its geometry details. **b,** Boundary setup of the time-domain meta-atom.

**Tab. S1 | Equivalent RLC parameter of the diode with different biasing voltages**

| Biasing Voltage (V) | C (pF) | R (Ω) | L (nH) |
| --- | --- | --- | --- |
| 0 | 2.31 | 4.51 | 0.70 |
| -6 | 0.62 | 3.84 | 0.70 |
| -9 | 0.45 | 3.44 | 0.70 |
| -21 | 0.22 | 2.13 | 0.70 |


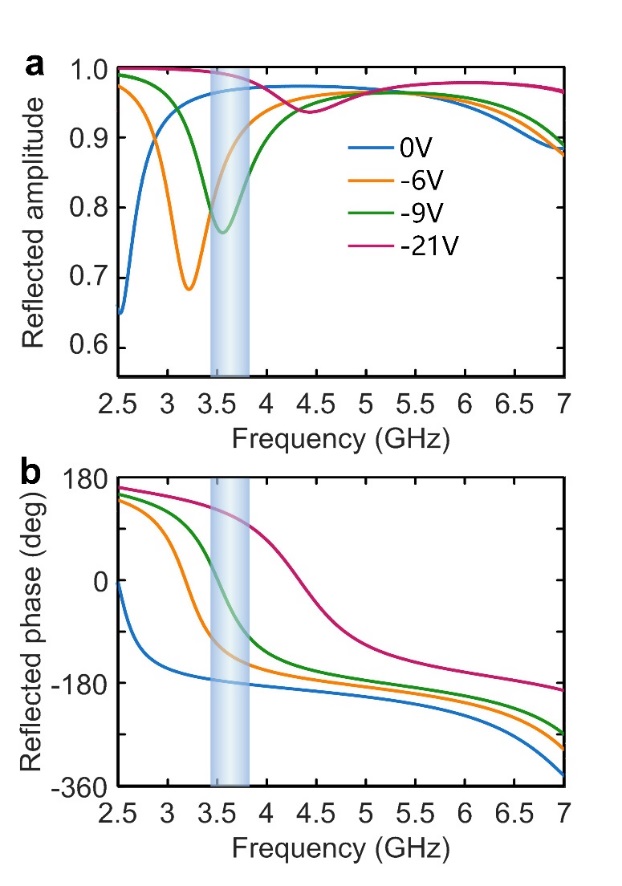


**Fig. S4 |** The simulated reflection amplitude (**a**) and phase (**b**) responses of the meta-atom under different biasing voltages, in which the blue region indicates the interested frequency region.

Theoretically, the 2-bit coding meta-atoms should have four phase states of 0°, 90°, 180° and 270° respectively. In the experiment, the four phase states are picked up when the meta-atom is biased under the voltages of 0V, -6V, -9V and -21V. The correspondence between the phase states in the theory and design for the 2-bit meta-atoms is listed below:

**Tab. S2 | The phase states for the 2-bit meta-atoms in the theory and design.**

| Digital Codes | Theoretical Phase (°) | Designed Phase (°) |
| --- | --- | --- |
| 00 | 0 | -175 |
| 01 | 90 | -105 |
| 10 | 180 | 16 |
| 11 | 270 | 110 |

**4. Experimental Setup of the Time-Domain Digital Coding Metasurface**

Photograph of the fabricated time-domain digital coding metasurface sample in experimental environment is illustrated in Fig. S5A, with the schematic of experimental setup shown in Fig. S5b. Here the standard printed circuit board (PCB) technology is used to fabricate the metasurface. The patterns on the top and bottom copper layers are obtained using the chemical etching process. The bare copper layers are covered with the photo-resist, and exposed to light through the photomask, so that the required tracks are retained as a result. In the measurements, a horn antenna is used as the excitation at a distance of 1.5m, and connected to a microwave signal generator (Agilent E8257D) in microwave anechoic chamber. The sample is mounted on a rotary table and fed by the horn antenna. When the sample and the source antenna are rotated simultaneously, we can monitor the angular distributions of the harmonics by a fixed receiving antenna 9m away, which is connected to a spectrum analyzer (Agilent E4447A). To clearly observe the spectrum characteristics of the echo signal, the resolution bandwidth (RBW) and the frequency span of the spectrum analyzer should be set small enough to separate the frequency components and find their accurate locations. Here we choose RBW and the frequency span to be 300Hz and 10MHz respectively in the experiment. The operation frequency of the transmitting antenna is 3.6GHz. An interface circuit board is designed to provide the dynamic biasing voltages under the programmable control of embedded system (NI Compact RIO platform).


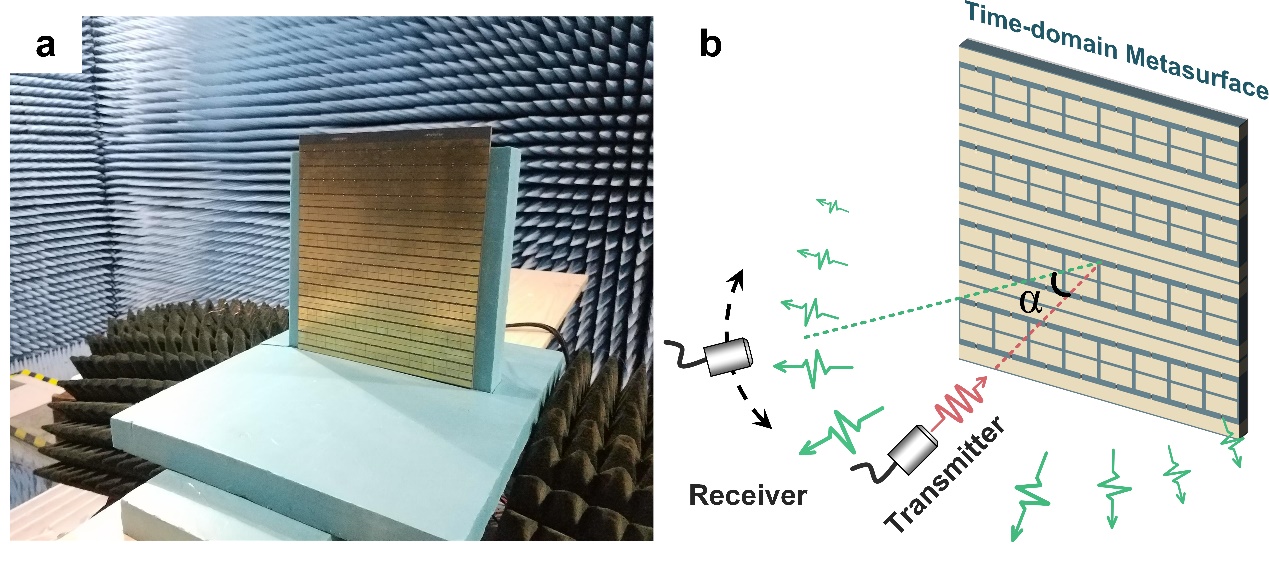


**Fig. S5 |** **a,** Photograph of the fabricated time-domain digital coding metasurface sample in experimental environment. **b,** Experimental setup to measure the spectral intensities and scattering patterns of the time-domain digital coding metasurface, where the transmitter generates electromagnetic waves in the normal direction, and the receiver is rotated along a semi-circular trajectory to monitor the field intensities. α is the receiving angle.

**5. Measured Reflection and Scattering Patterns**

The measured reflection spectra under the 1-bit digital coding sequence 01010101… at 3.6GHz with different pulse durations are illustrated in Fig. S6. We also measured the reflection spectra under different incident frequencies. Owing to the dispersion property from the meta-atom (as shown in Fig. S4a-b), the phase difference between the state ‘0’ (0 V) and ‘1’ (-9 V) would deviate from 180° when it is operated far from the central frequency 3.6GHz, leading to the deteriorated performance in nonlinearity generation. To find out the frequency dependence on the harmonic intensity, the time-domain coding metasurface is measured over the frequency range from 2GHz to 7GHz under the binary coding sequence 01010101…, and the reflection spectra are presented in Fig. S7a-d. Surprisingly, the metasurface appears to operate in a wide spectrum with distinct nonlinear phenomena. Theoretically, the harmonic generation always exists under the modulation of periodic PM signals, even when the phase difference $\Delta\phi$ of two states deviates significantly from 180°. Figure S7e-f illustrates the spectral intensities for the 0^th^-order and ±1^st^-order harmonics as the function of $\Delta\phi$. The suppression performance of the 0^th^-order harmonic starts to deteriorate rapidly due to the insufficient phase difference, which could be observed in the measured reflection spectra at 2, 5, and 7GHz in Fig. S7a, c, and d, respectively. We observe that the intensity of the 0^th^-order harmonic is much larger than that at 3.6GHz.

**
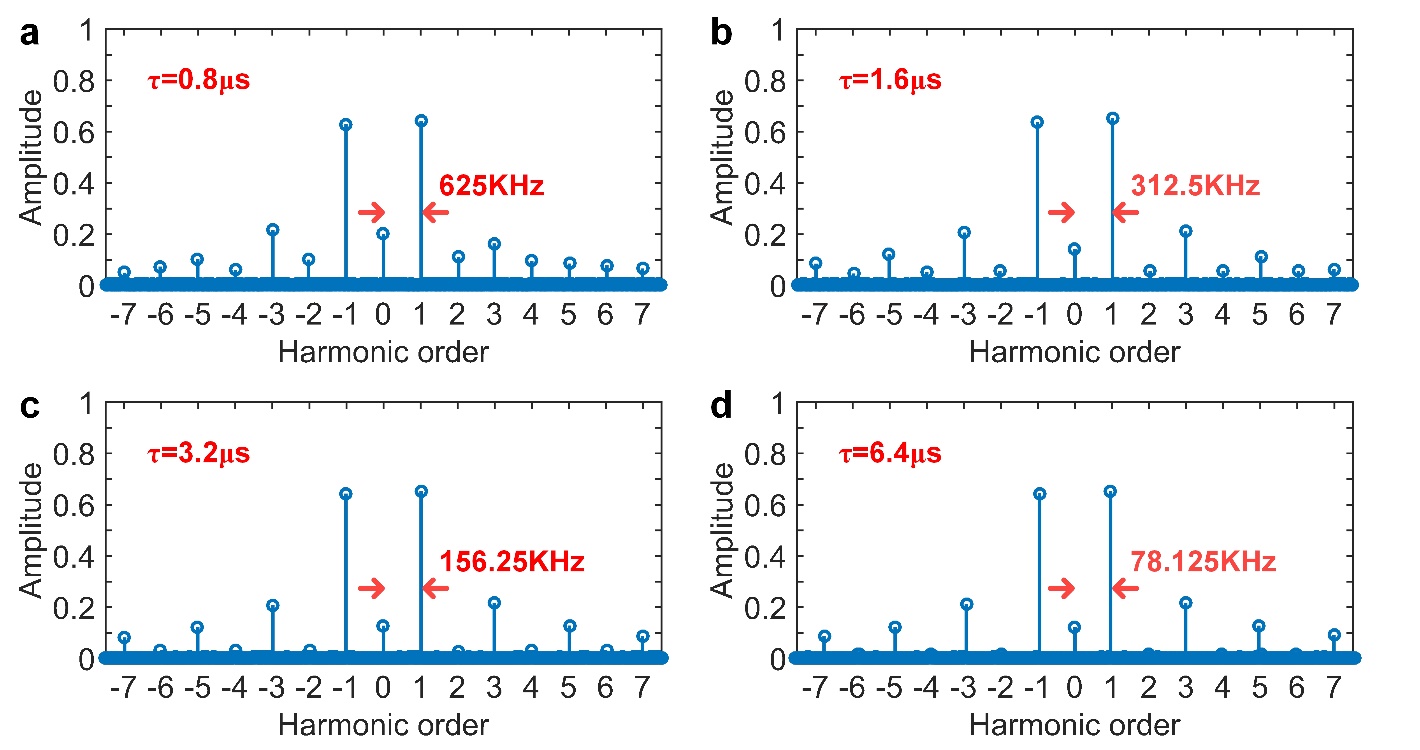
**

**Fig. S6 |** The measured spectral intensities of harmonics under the 1-bit coding sequence 01010101… at 3.6GHz with different pulse durations τ. **a,** τ=0.8μs. **b,** τ=1.6μs. **c,** τ=3.2μs. **d,** τ=6.4μs.

**
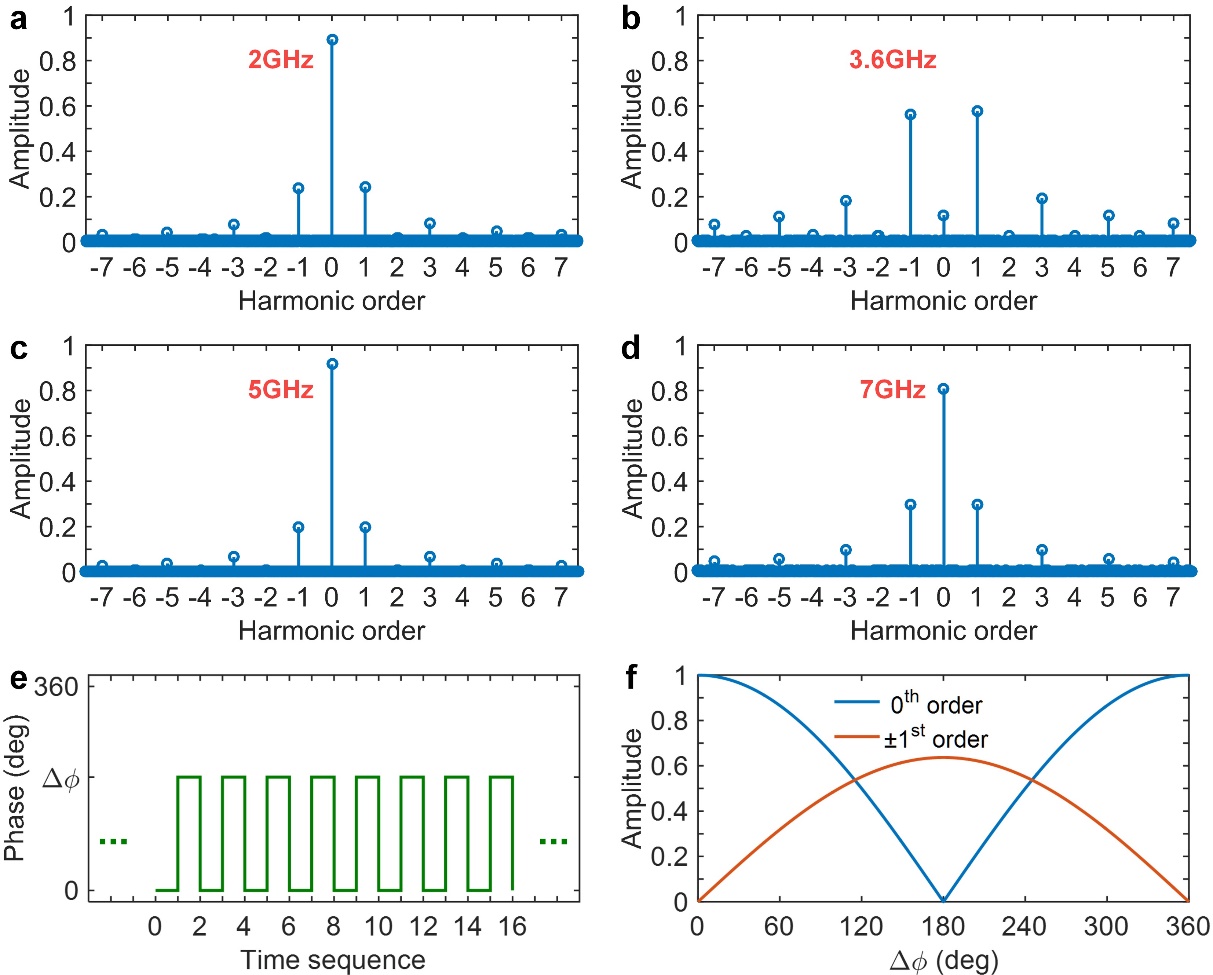
**

**Fig. S7 | a-d,** The measured spectral intensities of all harmonics under the periodic coding sequence 01010101… at 2GHz **a,** 3.6GHz b, 5GHz c, and 7GHz **d,** respectively. **e,** The phase modulation which switches between 0 and $\Delta\phi$ periodically. **f,** The theoretical spectral intensities for the 0^th^-order and ±1^st^-order harmonics as a function of $\Delta\phi$.

The efficiency of the frequency conversion is very important in practical applications, here we consider the 1-bit phase modulation with the coding sequence of 01010101..., τ=1.6μs, M=2, T=3.2μs, and 2-bit phase modulation with the coding sequence of 00-01-10-11-…, τ=1.6μs, M=4, T=6.4μs. The measured spectral intensities of the harmonics can be found in Fig. 3b and 3c respectively. The calculated and measured absolute conversion efficiencies from the input power to the power of different order harmonics are listed in Tabs. S3 and S4. It can be seen that both results are in good agreement, showing the effectiveness and correctness of this method.

**Tab. S3 | The absolute conversion efficiency of the metasurface under 1-bit phase modulation, with the coding sequence of 01010101...,**$\boldsymbol{\tau=1.6\mu s, M=2, T=3.2\mu s}$**.**

| Harmonic order | Measured (%) | Theoretical (%) | Error (%) |
| --- | --- | --- | --- |
| $-$7^th^ | 0.57 | 0.69 | -0.12 |
| $-$6^th^ | 0.17 | 0.00 | 0.17 |
| $-$5^th^ | 1.18 | 1.35 | -0.17 |
| $-$4^th^ | 0.22 | 0.00 | 0.22 |
| $-$3^rd^ | 3.32 | 3.76 | -0.44 |
| $-$2^nd^ | 0.25 | 0.00 | 0.25 |
| $-$1^st^ | 31.61 | 33.80 | -2.19 |
| 0^th^ | 1.55 | 0.28 | 1.27 |
| +1^st^ | 33.12 | 33.80 | -0.68 |
| +2^nd^ | 0.25 | 0.00 | 0.25 |
| +3^rd^ | 3.47 | 3.76 | -0.29 |
| +4^th^ | 0.26 | 0.00 | 0.26 |
| +5^th^ | 1.02 | 1.35 | -0.33 |
| +6^th^ | 0.24 | 0.00 | 0.24 |
| +7^th^ | 0.32 | 0.69 | -0.37 |

**Tab. S4 | The absolute conversion efficiency of the metasurface under 2-bit phase modulation, with the coding sequence of 00-01-10-11-…,**$\boldsymbol{\tau=1.6\mu s, M=4, T=6.4\mu s}$

| Harmonic order | Measured (%) | Theoretical (%) | Error (%) |
| --- | --- | --- | --- |
| $-$7^th^ | 1.74 | 1.47 | 0.27 |
| $-$6^th^ | 0.33 | 0.00 | 0.33 |
| $-$5^th^ | 0.33 | 0.00 | 0.33 |
| $-$4^th^ | 0.23 | 0.00 | 0.23 |
| $-$3^rd^ | 9.67 | 8.03 | 1.64 |
| $-$2^nd^ | 0.93 | 0.03 | 0.90 |
| $-$1^st^ | 0.46 | 0.08 | 0.38 |
| 0^th^ | 0.41 | 0.08 | 0.33 |
| +1^st^ | 66.47 | 72.27 | -5.80 |
| +2^nd^ | 0.18 | 0.03 | 0.15 |
| +3^rd^ | 0.06 | 0.01 | 0.05 |
| +4^th^ | 0.23 | 0.00 | 0.23 |
| +5^th^ | 0.48 | 2.89 | -2.41 |
| +6^th^ | 0.07 | 0.00 | 0.07 |
| +7^th^ | 0.06 | 0.00 | 0.06 |

Besides the reflection spectra intensities, we have also measured the scattering patterns of the ±1^st^-order harmonics on the H-plane under the periodic coding sequence 11-10-01-00-…, as illustrated in Fig. S8.


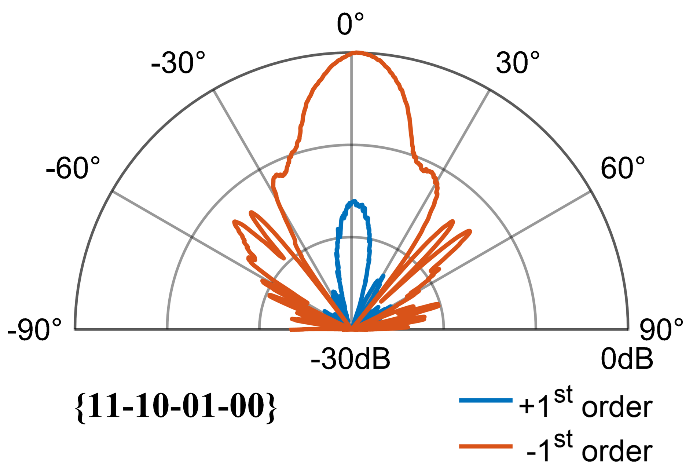


**Fig. S8 |** The measured H-plane scattering patterns of the +1^st^ (the blue line) and -1^st^ (the red line) order harmonics under the periodic coding sequence of 11-10-01-00-….

In addition, by introducing both space and time gradient into the metasurface, it will open up a new avenue to engineer the propagation behaviors of the higher order harmonics. For example, two cases are considered in Fig. S9a, where the whole metasurface is controlled by the coding sequence S_1_ (00-01-10-11-…), or its four columns are alternatively controlled by the coding sequences S_1_ (00-01-10-11-…) and S_2_ (10-11-00-01-…). Note that S_1_ and S_2_ actually stand for the same modulation signals with the time difference equal to half of the period, so that they will lead to the same harmonic intensities but opposite phases for neighboring columns.

Fig. S9c shows that in Case I, both the $\pm$1^st^ order harmonics are beamed along the normal direction with distinct intensities, due to the anisotropic harmonic spectra in Fig. S9b. However, in Case II, we can control the bending direction of the scattering beam at normal incidenceaccording to the generalized Snell’s law. The theoretical bending angle of +1^st^ order harmonic in Case II is $\pm34^{\circ}$ from the antenna theory [1], and the measured result is $\pm34^{\circ}$ as shown in Fig. 9d (red line), originating from the spatial phase difference between two periodic coding sequences. This technique can be further exploited to manipulate the propagation of higher-order harmonics.

**
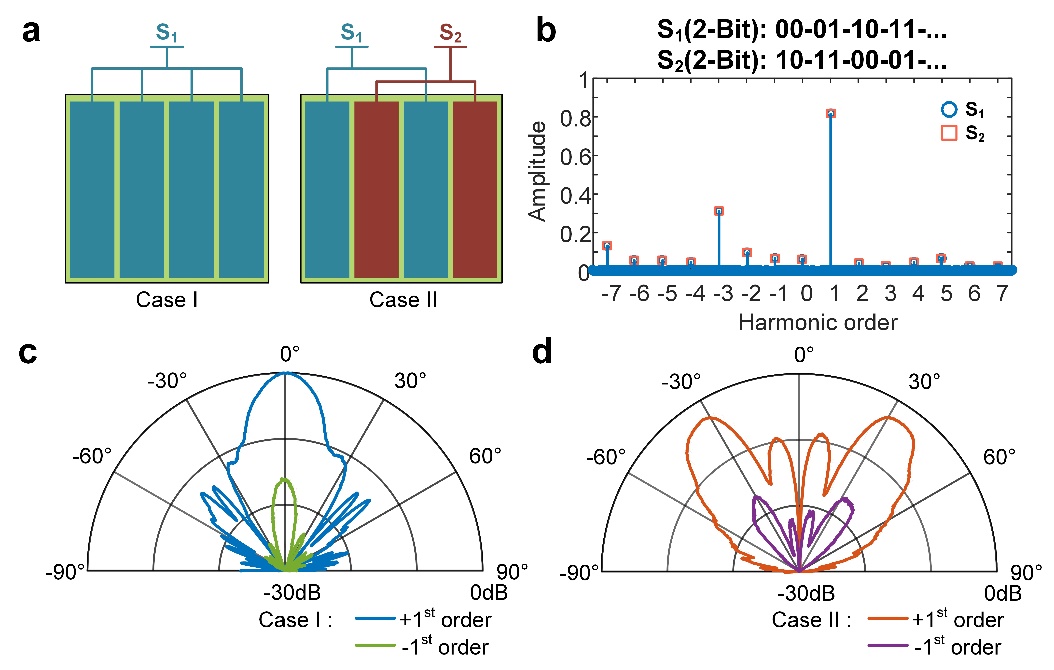
**

**Fig. S9 | a,** Schematic of the metasurface under 2-bit phase modulation. The four columns are modulated by the coding sequence S_1_ (00-01-10-11-…) in Case I or the coding sequences S1 (00-01-10-11-…) and S_2_ (10-11-00-01-…) alternatively in Case II. **b,** The measured spectral intensities of the harmonics in Case I when modulated by S_1_ and S_2_. **c,** The measured H-plane scattering pattern of the +1^st^ (blue) and -1^st^ (green) order harmonics in Case Ⅰ. **d,** The measured H-plane scattering pattern of the +1^st^ (orange) and -1^st^ (purple) order harmonics in Case Ⅱ.

**6. Necessity of Digital Modulation Instead of Analog Modulation**

Analog amplitude or phase modulation could actually enhance the spectral purity with excellent suppression of spurs in the echo signal from the metasurface. However, in practice the digital modulation is more advantageous due to the following reasons. Firstly, the digital modulation can greatly simply the control circuit of the metasurface since only finite phase/amplitude states are required during the modulation, and thus becomes more suitable for high-speed applications. Secondly, the analog phase modulation usually needs full-phase range to increase the nonlinear conversion rate, which is hard to achieve for the proposed metasurface by tuning the varactor at the presence of material loss. However, discrete phase states of the digital modulation are much easier to accomplish in reality, and therefore lower the barrier of element design.

Note that the binary meta-atoms usually do not have equal amplitudes as shown in Fig. S4a. To illustrate the amplitude-influence of the overall phase modulation performance, we have simulated the spectral intensities of the harmonics under the coding sequence of 01010101..., in which the element reflection amplitudes are 1 and 1-$\delta$ respectively for 1 bit meta-atom with 0° and 180° binary phases. Here$\delta$is a positive value smaller than unity. When$\delta=0,$ the spectral intensity distributions are the same as that in Fig. S10a. However, with the increase of$\delta$ from 0.2 to 0.8 (see Fig. S10b-S10d), we clearly observe the rapid growth of the 0th order harmonic, since the amplitude difference will lead to the nonzero average value of the reflection coefficient, although the phases are alternatively switched between 0° and 180°. Hereby in order to keep large conversion efficiency, $\delta$ should be small in the design.


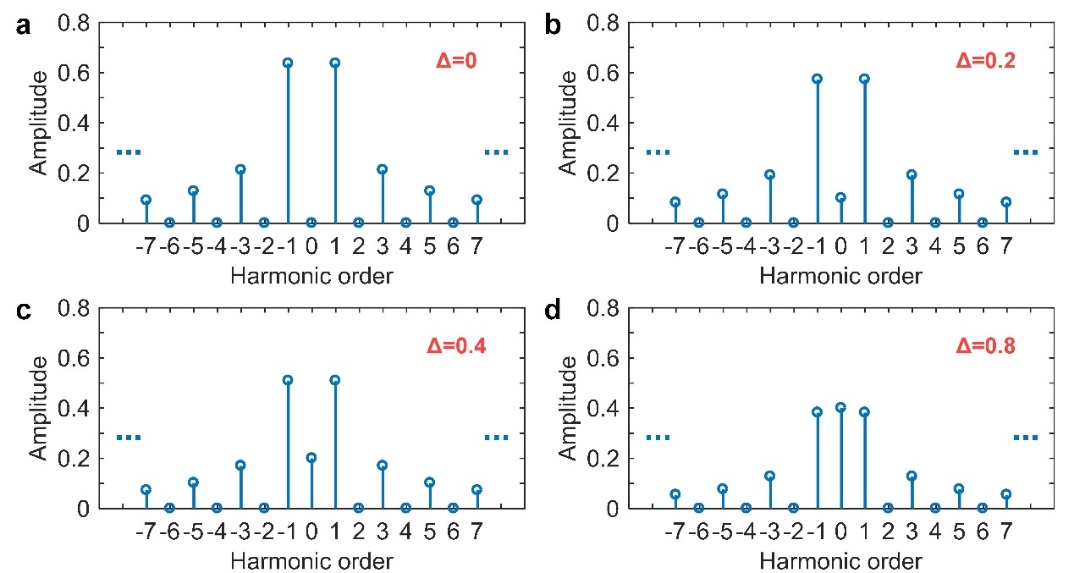


**Fig. S10 |** Simulated spectral intensities of the harmonics under the coding sequence of 01010101..., in which the element reflection amplitudes are 1 and 1-$\delta$ respectively for 1 bit meta-atom with 0° and 180° binary phases. Here$\delta$is 0 (**a**), 0.2 (**b**), 0.4 (**c**) and 0.8 (**d**) respectively.

**7. Traditional Wireless Communication System**

Figure 5a illustrates a schematic of the conventional super-heterodyne wireless communication system, whose architecture makes great difference from the metasurface-based system in the RF portion described in Figure 4a. The conventional wireless communication system usually consists of a digital baseband and analog RF modules, which are connected by the digital-to- analog converter (DAC). The baseband module serves as the digital signal processing center and is responsible for the implementation of various baseband signal processing algorithms; while the RF module is responsible for the up/down-conversion, carrier signal modulation, and power boosting, and hence contains a series of passive and active microwave components, including mixers, filters, low noise amplifiers, and power amplifiers, etc. The main task of the RF module is the manipulation of the carrier signal based on the message to be transmitted.

This function can be implemented by the proposed time-domain digital coding metasurface, which can directly manipulate the incident electromagnetic waves in free space, and embed the message into the reflected waves with various coding sequences. Here we take a BFSK system for example. The message could be encoded as the bit steam like ‘01101001…’ with the bit ‘1’ and ‘0’ represented by the +1st and -1st order harmonic frequencies respectively by adopting opposite coding sequences 00-01-10-11… and 11-10-01-00…, as shown in Fig. 3c and 3d. Therefore, the baseband message is embedded into the reflected waves and could be handled by the receiving system.

The bit error ratio (BER) of this system is dependent on the signal noise ratio (SNR) at the receiver terminal, which is related to the transmitting power, the receiving angles and distances. We have summarize these relationships in Fig. S11. For large transmitting power, the SNR is quite large and the BER is especially low in the experiment, which seems independent on these factors (Fig. S11). For small transmitting power, the SNR becomes smaller and smaller with the increase of the distance and incident angles, so the BER grows rapidly during the data transmission.


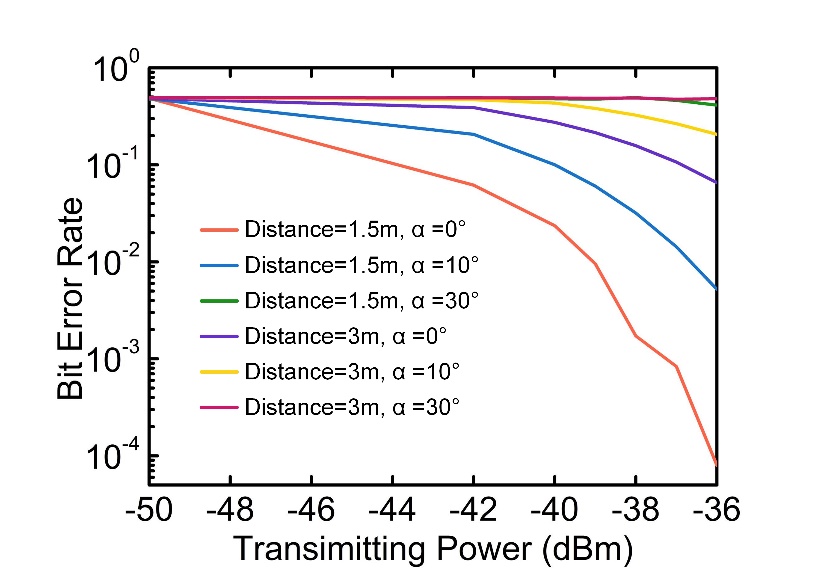


**Fig. S11** | Dependence of the bit error rate of the proposed BFSK system on the transmitting power under different distances and incident angles.

**8. SOM Videos**

The processes of picture transmission through the new BFSK system based on the time-domain digital coding metasurface are recorded in SOM Videos 1-3. The message, a color picture of the logo for Southeast University, is transmitted through the new BFSK transmitter, which is received by an SDR receiver placed 6.25 m away from the BFSK transmitter.

**Video 1.** When the normal of metasurface is directed to the receiver ($\alpha=0^{\circ}$), the picture transmitted by the new BFSK transmitter is received and successfully recovered by receiver, validating the performance and feasibility of our system.

**Video 2.** To verify the robustness of the system, another experiment is carried out, where the new BFSK transmitter is swinging continuously between $\alpha=-30^{\circ}$ and $\alpha=30^{\circ}$ during the message transmission. Under this circumstance, the message is received and recovered by the receiver perfectly, indicating that the new BFSK system could cover the region about at least 60^o^ azimuth.

**Video 3.** To prove the characteristic of anti-interference property of the system, an interference source is placed close to the metasurface, generating the interference signal with very high power intensity. The operation frequencies for the BFSK system are 3.6GHz±312.5KHz. In the experiment, the frequency for interference signal changes step by step from 3.6GHz+10MHz, 3.6GHz+2MHz, to 3.6GHz+550KHz, gradually approaching the operation frequency (3.6GHz+ 312.5KHz) of the new BFSK system. The experiment result shows that the transmitted signal from the BFSK system could still be received and recovered.

In conclusion, the proposed new BFSK system could work properly in some critical environments, and might find applications in the wireless transmission system like base station system.

**References**

1 Kraus, J, D, Marhefka, R, J. *Antennas for all applications*. McGraw-Hill, 2003.
